# Insecure Attachment and Depressive Symptoms among a Large Sample of Chinese Young Adults: The Mediating Role of Positive and Negative Self-Compassion

**DOI:** 10.3390/bs14030238

**Published:** 2024-03-15

**Authors:** Shuhan Yang, Yizhen Ren, Xi Song, Jie Ge, Yu Peng

**Affiliations:** 1 Faculty of Education, Yunnan Normal University, Kunming 650500, China; 2034010001@ynnu.edu.cn; 2 Faculty of Psychology, Beijing Normal University, Beijing 100875, China; 201931061008@mail.bnu.edu.cn; 3 Students Mental Health Education & Counseling Center, Kunming University of Science and Technology, Kunming 650500, China; songxi@kust.edu.cn (X.S.); 20110062@kust.edu.cn (J.G.); 4 Faculty of Social Sciences & Liberal Arts, UCSI University, Kuala Lumpur 56000, Malaysia

**Keywords:** attachment anxiety, attachment avoidance, positive self-compassion, negative self-compassion, depressive symptoms

## Abstract

Objective: The present study aimed to explore the relationship between insecure attachment (attachment anxiety and avoidance) and depressive symptoms in Chinese young adults as well as the mediating roles of positive and negative self-compassion. Methods: An online survey was administered among college students in mainland China. This involved their completion of questionnaires encompassing socio-demographic details, attachment anxiety, attachment avoidance, self-compassion, and depressive symptoms. Results: Attachment anxiety and avoidance exhibited connections with depressive symptoms through increased negative self-compassion and decreased positive self-compassion. Attachment anxiety was associated with depressive symptoms primarily through the mediating effect of negative self-compassion. Conversely, attachment avoidance was related to depressive symptoms mainly through the mediating role of positive self-compassion. Conclusions: Attachment anxiety exerted a slightly stronger influence on negative self-compassion, whereas attachment avoidance exhibited a more prominent impact on positive self-compassion. Despite these differences, both attachment styles were comparable in their overall influence on depressive symptoms. This revelation provides fresh insights into the relationship between insecure attachment and depressive symptoms among young adults, underscoring the importance for intervention program development.

## 1. Introduction

The prevalence of severe depression among Chinese college students is high, and navigating interpersonal relationships poses a significant developmental challenge within this population [1,2]. Hence, this study concentrates on interpersonal factors that contribute to depressive symptoms in college students, aiming to offer more specific intervention recommendations [1,3]. Specifically, insecure attachment, including attachment anxiety and avoidance, which mirror individuals’ insecure internal representations of themselves, others, and their interactions, could potentially have an adverse impact on depressive symptoms among college students [4,5]. From an attachment theory perspective [5], insecure attachment divides into attachment anxiety and attachment avoidance, delineated by contrasting perceptions of the self and others [6]. This dynamic significantly impacts the emergence of depressive symptoms [7]. Moreover, insecure attachment affects an individual’s self-perception [5,8], influencing the development of depressive symptoms by shaping how individuals treat and relate to themselves, which is reflected in their degree of self-compassion [9,10]. Hence, the present study investigated the connections between insecure attachment (anxiety and avoidance) and depressive symptoms among young adults while also examining the mediating impacts of positive and negative self-compassion.

### 1.1. Insecure Attachment and Depressive Symptoms

Depressive symptoms in young individuals represent a substantial worldwide public health issue [1]. Research indicates that the prevalence of depression is 29.4%, with 7% to 8% of university students in the six ASEAN countries exhibiting suicidal tendencies [3]. Moreover, the worldwide repercussions of the COVID-19 pandemic have amplified depression levels among young adults universally, resulting in an increase in the severity of depressive symptoms [1]. Navigating interpersonal relationships during the college transition period poses a substantial developmental challenge, with established evidence indicating the critical role of such relationships in influencing depressive symptoms [2,11].

Teichman et al. [12] introduced a reciprocal depression model, highlighting that distortions and dysfunctions in cognition, emotions, and behaviors within meaningful relationships play a role in both the formation and persistence of depressive symptoms. From this perspective, depression is seen as a consequence of interpersonal relationship processes. The theory emphasizes that the interaction between cognition, emotion, and behavior reinforces depression at both the internal and interpersonal levels [12]. Our specific focus is on insecure attachment, which involves adverse mental representations of oneself, others, and the social world resulting from repeated interactions with caregivers and important attachment figures. Drawing on attachment theory [5], our proposition was that individuals exhibiting insecure attachment orientations often harbor adverse perceptions regarding their relationships with others. Consequently, we expect that insecure attachment styles would exhibit a positive association with depressive symptoms. Specifically, individuals characterized by a secure attachment style are notably inclined to form robust and supportive interpersonal relationships [13,14] characterized by mutual understanding and assistance [15], which can help alleviate mental health problems [16]. Conversely, individuals demonstrating an insecure attachment style might display elevated levels of anxiety or avoidance behaviors in their interpersonal relationships, potentially leading to conflicts and tension, subsequently contributing to the emergence of depressive symptoms [16,17].

Attachment theory suggests that during repeated interactions with caregivers and important attachment figures, individuals develop mental representations of themselves, others, and the social world [5,8,18]. Insecure attachment representation was developed from two attachment working models: the self-model and other-model [5,8,19]. Specifically, insecure attachment is divided into two dimensions: attachment anxiety and avoidance. On the one hand, attachment anxiety involves negative self-modeling, wherein individuals with this attachment style require continuous acceptance from others to maintain positive self-concern [6] due to excessive fear of abandonment [20]. Conversely, attachment avoidance is defined by negative other-modeling, causing individuals to evade close contact with others because of the expectation of unfavorable consequences [6,21]. In other words, a negative self-pattern is similar to attachment anxiety, while a negative other-pattern is comparable to attachment avoidance [6,22]. Attachment anxiety pertains to varying levels of sensitivity to abandonment, whereas attachment avoidance indicates distinctions in intimacy and emotional expression [23,24]. Typically, an individual’s attachment style impacts their interactions with loved ones and partners [25].

Recent research has explored the intricate relationship between insecure attachment styles and depression. Anxious attachment tends to amplify the perception of threat and lack of control over distress, whereas avoidant attachment tends to deny negative effects, both of which contribute to increased distress and exacerbate depressive symptoms [4,26]. However, it is essential to emphasize the significant impact of specific attachment styles on depressive symptoms. A meta-analysis by Zheng et al. [26] highlighted that attachment anxiety displays a stronger correlation with depressive symptoms compared to attachment avoidance, which shows a relatively weaker connection. Those individuals with heightened attachment anxiety or avoidance tendencies are more susceptible to experiencing depressive symptoms, especially when attachment anxiety is intensified [26]. Therefore, given the diverse characteristics of various insecure attachment styles, this study examines the relationship between different insecure attachment styles and depressive symptoms.

### 1.2. The Mediating Roles of Positive Self-Compassion and Negative Self-Compassion

To enhance intervention programs and illuminate potential mediating mechanisms between insecure attachment and depressive symptoms (DS) in young adults, a deeper grasp of attachment theory is essential. From the perspective of attachment theory, attachment orientations reflect internalized evaluations and expectations of the self and important others [8]. In this sense, insecure attachment may impact the way individuals connect to themselves and influence their self-worth and self-evaluation [10], manifesting within the construct of self-compassion [9,10]. Self-compassion denotes an individual’s ability for self-tolerance and kindness during adversity, encompassing three fundamental components: self-kindness, common humanity, and mindfulness [27]. Self-compassion can be delineated into positive and negative dimensions [28]. Specifically, “positive self-compassion” encompasses treating oneself with kindness, understanding, and forgiveness during challenging moments or setbacks, consisting of three key elements: self-kindness, common humanity, and mindfulness [29,30,31]. In contrast, “negative self-compassion” involves self-criticism, self-blame, and harsh self-assessment, resulting in self-judgment, isolation, and an exaggerated sense of self-identification. [29,30,31]. These dimensions offer insight into the nuanced aspects of self-compassion, encompassing both positive and negative reactions to personal adversities.

There is a suggestion that early attachment experiences could influence self-compassion, which, in turn, correlates with mental health [9,10]. Individuals with diverse attachment styles typically have distinct perceptions of themselves [32]. When caregivers consistently meet a child’s needs, the child feels comforted and supported, internalizing positive self-perceptions and relational patterns [13]. Conversely, inadequate caregiver responses can lead individuals to believe they are unworthy of support or that others are unreliable, resulting in negative self and relational patterns [13]. Prior studies have shown that insecure attachment significantly predicts a generally reduced level of self-compassion among young adults [33,34,35].

Individuals experiencing attachment anxiety frequently tend to cultivate a pessimistic self-perception, wherein they often engage in self-criticism rather than self-care [34,36]. Their tendency is to seek external validation and attention from the world around them [20] makes it challenging for them to draw upon their internal resources for fostering positive self-compassion [37]. The profound fear of abandonment significantly influences the negative self-image of individuals characterized by attachment anxiety. For instance, those with attachment anxiety might gradually form the belief that they are “bad” and undeserving of love, stemming from their excessive concerns about abandonment [38]. Their pursuit of perfectionism often leads to an intolerance of personal imperfections, fostering a tendency toward harsh self-judgment [39], self-imposed isolation [40], and an over-identification with negative emotions [24,41]. Hence, individuals experiencing attachment anxiety might demonstrate reduced positive self-compassion and increased negative self-compassion.

Individuals exhibiting attachment avoidance often maintain both negative and positive self-views [22,32,42]. Opposite to the core of self-compassion, which embodies inclusive and gentle self-acceptance during distress situations [43], those with attachment avoidance exhibit heightened defense mechanisms that deter vulnerability [44]. Simultaneously, they harbor a belief that they are not accepted [10]. Consequently, individuals displaying high levels of attachment avoidance may impose stringent expectations on themselves, aiming to rely on their own capacities rather than seeking support from others, which can lead to reduced self-compassion [34,42,45]. They tend to demonstrate less self-soothing behavior but show increased signs of depression and defensiveness [46]. Therefore, individuals displaying attachment avoidance may exhibit a positive correlation with negative self-compassion and a negative correlation with positive self-compassion [33].

Recent studies frequently highlight the connection between self-compassion and depressive symptoms [47,48,49]. Self-compassion alleviates negative emotions through positive regulatory strategies such as self-kindness, mindfulness, and common humanity [27,50,51]. Therefore, self-compassion is an effective way to keep yourself from immersing yourself in negative emotions [30]. From the perspective of emotional regulation, self-compassion can enable individuals to engage in cognitive reappraisal and become aware of and understand their current emotional state, thereby alleviating depression. [48]. Additionally, earlier studies indicated a stronger correlation between depressive symptoms and the negative aspects of self-compassion, while the link between depressive symptoms and positive dimensions of self-compassion appeared relatively weaker [52]. Nevertheless, the need for further exploration and clarification lies in understanding whether and how positive and negative self-compassion mediate the relationships between attachment styles (anxiety and avoidance) and depressive symptoms.

### 1.3. The Current Study

In this study, a mediation model was constructed to examine how insecure attachment predicts depressive symptoms through self-compassion among college students. We hypothesize that attachment anxiety and attachment avoidance could predict the presence of depressive symptoms. Additionally, our hypothesis proposes that both positive and negative self-compassion would significantly mediate the predictive relationships between insecure attachment and depressive symptoms.

## 2. Materials and Methods

### 2.1. Participants

This study collected 44,788 valid questionnaires. Among the surveyed college students, the average age was 21.40 years (standard deviation = 2.38), ranging from 18.0 to 30.0 years. Of these students, 29,338 (65.5%) were male, while 15,450 (34.5%) were female. Furthermore, the study included 31,999 (71.4%) undergraduate students and 12,789 (28.6%) graduate students. Undergraduate programs typically span four years, whereas master’s programs typically span three years. Additionally, data on student numbers corresponding to their majors were collected. Participants’ enrollment years ranged from 2016 to 2022, with no relocations occurring during the study period. For detailed demographic information, please refer to Table 1.

### 2.2. Measures

Sociodemographic characteristics. Participants were invited to report their age, gender (male = 1 and female = 2), the only-child status (the only child = 1 and not the only child = 2), family type, and residence (rural = 1, town = 2, and urban = 3).

Adult Attachment Scale (AAS). The AAS was developed in 1990 by Collins and Read to assess insecure attachment types [53]. The scale comprises 18 items designed to evaluate two dimensions: attachment anxiety and avoidance. Respondents rated each item using a 5-point Likert scale ranging from 1 (not at all) to 5 (very much). Higher scores on either dimension reflected increased levels of attachment anxiety or avoidance. This scale was validated [54] in 2012 by Qu and Gan and is commonly utilized among Chinese young adults. The Cronbach’s α for the Chinese version of the AAS was 0.72 [55]. The Cronbach’s α coefficients for attachment anxiety and avoidance in this study were 0.855 and 0.731.

Short Form of Self-Compassion Scale (SCS-SF). Using the 12-item Self-Compassion Scale (SCS) by Neff, participants evaluated six dimensions: self-kindness, self-judgment, common humanity, isolation, mindfulness, and over-identification. They responded on a 5-point scale ranging from “almost never” to “almost always”. The SCS-SF items were derived from the original 26-item version. This scale was validated in 2023 by Huang et al. [56] and is widely used to gauge self-compassion in Chinese populations [57]. The Cronbach’s α for the Chinese version of the SCS-SF was 0.77 [56]. The Cronbach’s α coefficient for positive self-compassion was 0.824, and for negative self-compassion, it was 0.731 in the current study.

Beck Depression Inventory (BDI). The BDI is a widely used self-rating scale for depression [58] and consists of 21 statements graded by severity, with each item scored from 0 to 3 points. Total scores range from 0 to 63, where scores between 14 and 19 suggest a mild mood disorder, and a score of 20 is the clinical cutoff for diagnosing depression. Higher scores on the scale denote more severe depression. Yang et al. demonstrated good reliability and validity of the scale in a Chinese sample in 2014 [59]. The Cronbach’s α for the Chinese version of the BDI was 0.89 [59]. The Cronbach’s α coefficient was 0.889 in the current study.

### 2.3. Procedures and Data Analysis

Participants took part in the research by engaging in an online survey. Teachers utilized a multi-channel approach, strategically placing posters throughout the college and leveraging WeChat and QQ groups to actively promote recruitment. Prospective participants were encouraged to conveniently access the questionnaire by scanning QR codes featured on the posters or within the designated WeChat and QQ groups. The questionnaire included socio-demographic characteristics, attachment anxiety, attachment avoidance, self-compassion, and depressive symptoms. Enrolled students were requested to sign an e-consent form before completing the questionnaire. The study obtained approval from the institutional review board at the author’s affiliated institution.

Descriptive statistics and correlation analyses were performed using SPSS 26.0. The study employed the structural equation model (SEM) in Mplus 8.4 to examine how attachment anxiety and avoidance predict depressive symptoms. Age and gender were considered control variables in the SEM. Bootstrap analysis, utilizing 2000 replicates, evaluated the 95% confidence intervals for the mediating effects of positive and negative self-compassion within the SEM.

## 3. Results

### 3.1. Preliminary Analysis

Table 2 presents the means, standard deviations, and Pearson correlations among the study variables. In this study, the mean score for attachment anxiety was 14.564 (*SD* = 4.817), and the mean score for attachment avoidance was 30.009 (*SD* = 5.887). Moreover, the mean score for positive self-compassion was 22.211 (*SD* = 4.014), and the mean score for negative self-compassion was 15.949 (*SD* = 4.331). Additionally, the mean score for depressive symptoms was 4.991 (*SD* = 6.503). This was consistent with previous studies [60,61,62]. Attachment anxiety and attachment avoidance exhibited negative correlations with positive self-compassion and positive correlations with negative self-compassion. Moreover, positive self-compassion demonstrated a negative correlation with depressive symptoms, whereas negative self-compassion displayed a positive correlation with depressive symptoms.

### 3.2. The Mediation Model

**Figure 1:**
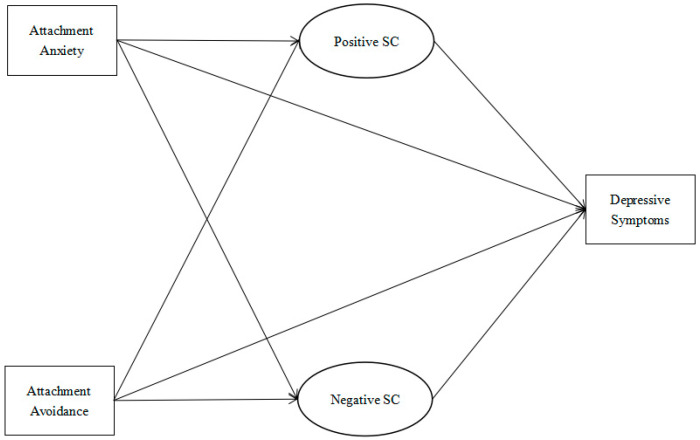
The hypothesized mediation model.

**Figure 2:**
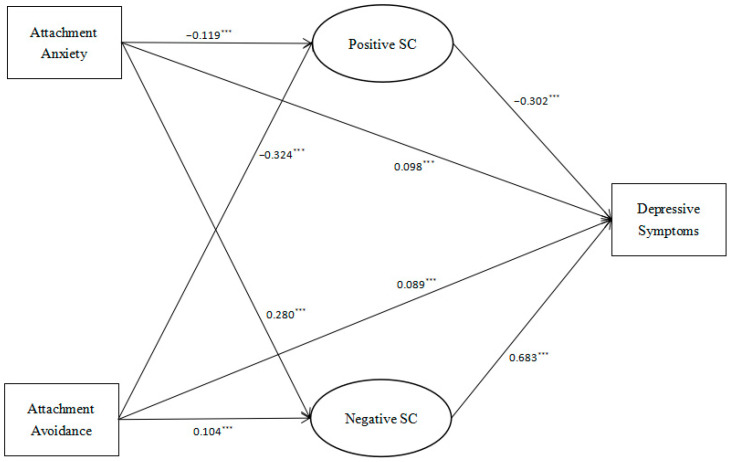
The mediation model for the total sample. Note. *** *p* < 0.001.

Upon controlling for various variables, the examined model achieved acceptable fit indices (RMSEA = 0.062, CFI = 0.953, TLI = 0.937, SRMR = 0.050). The specifics of the mediation model are outlined in Table 3, while the particular relationship paths are depicted in Figure 1 and Figure 2. Table 3 demonstrates that the 95% bootstrap confidence interval for the mediation effects of positive and negative self-compassion did not encompass zero, indicating that these mediation effects were all statistically significant.

In the overall model, attachment anxiety (*β* = −0.119, *p* < 0.001) and avoidance (*β* = −0.324, *p* < 0.001) negatively predicted positive self-compassion, which then negatively predicted depressive symptoms in young adults (*β* = −0.302, *p* < 0.001). Additionally, attachment anxiety (*β* = 0.280, *p* < 0.001) and avoidance (*β* = 0.104, *p* < 0.001) positively predicted negative self-compassion, which then positively predicted depressive symptoms in young adults (*β* = 0.683, *p* < 0.001). Furthermore, attachment anxiety and avoidance positively predicted depressive symptoms (*β* = 0.098, *p* < 0.001; *β* = 0.089, *p* < 0.001).

## 4. Discussion

This research formulated a mediation model to investigate the correlation between insecure attachment and depressive symptoms among Chinese young adults. This model employed positive and negative self-compassion as mediators in the analysis. The findings indicated significant links between insecure attachment and depressive symptoms, demonstrating notable mediation through both positive and negative self-compassion. Specifically, attachment anxiety and avoidance positively predicted depressive symptoms through increased negative self-compassion and decreased positive self-compassion. The influence of attachment anxiety on depressive symptoms was comparable to that of attachment avoidance. However, attachment anxiety showed a more significant effect on negative self-compassion than attachment avoidance did, whereas attachment avoidance exhibited a stronger influence on positive self-compassion compared to attachment anxiety.

### 4.1. The Mediating Effect of Positive and Negative SC

The present research identified a correlation between attachment anxiety and avoidance with depressive symptoms. This association was found to be mediated by both positive and negative self-compassion, aligning with prior research results [9]. Self-compassion refers to one’s capacity to display tolerance and compassion toward oneself amid moments of distress or hardship [27,30], characterized by two dimensions: positive and negative [29]. The results revealed that attachment anxiety and avoidance positively predicted negative self-compassion and negatively predicted positive self-compassion.

Attachment styles shape individuals’ perspectives of self [32]. Insecure attachment cultivates a sense of discomfort, facilitating negative self-perception and leading to feelings of unworthiness and mistrust [13]. Therefore, insecure attachment predicts lower self-compassion in young adults [33,63,64]. Those with attachment anxiety often harbor negative self-views and rely on external validation, struggling to cultivate internal self-compassion [36,37]. For individuals with high attachment anxiety, fear of abandonment fuels self-deprecating beliefs, perfectionism, and harsh self-judgment [38,39], leading to isolation and excessive identification with negative emotions [24,41]. Attachment avoidance manifests itself as varied self-views, exhibiting defensive mechanisms and a reluctance to acknowledge vulnerability [22,44]. Individuals with high attachment avoidance may demand self-reliance, leading to reduced positive self-compassion [34,42,45] and increased depression [46]. Consequently, attachment avoidance typically shows a positive correlation with negative self-compassion and a negative correlation with positive self-compassion [33].

Furthermore, the link between attachment anxiety and negative self-compassion demonstrates a notably stronger connection than that observed between attachment avoidance and negative self-compassion. This difference might stem from the traits found in individuals with attachment anxiety, often characterized by a tendency towards negative self-perceptions [6,38]. People with high attachment anxiety often exhibit increased self-criticism, feelings of isolation, and a strong inclination to strongly associate themselves with their negative emotions and distress [10,39,40]. However, individuals with attachment avoidance do not necessarily have a negative view of themselves [32]. They will use depressive and divisive defense mechanisms, downplay emotional experiences, and avoid seeking support from others [65,66]. Consequently, individuals characterized by attachment avoidance tend to display a weaker association with negative self-compassion compared to individuals with attachment anxiety.

In contrast, attachment avoidance showed a stronger correlation with positive self-compassion. This could be attributed to individuals with attachment avoidance actively avoiding reliance on others and experiencing greater difficulty in forming meaningful connections [10,45]. People experiencing attachment anxiety tend to harbor self-disapproval but still value connections with others [34,45]. Consequently, individuals exhibiting attachment avoidance might exhibit reduced self-empathy, a diminished capacity to connect with themselves, and difficulty extending self-kindness derived from supportive relationships with others [10]. Conversely, individuals with attachment avoidance often display greater self-reliance and a defensive shield against potential harm from others, a trait less observed in the attachment-anxious cohort [10]. Hence, individuals with attachment avoidance display a heightened link to positive self-compassion.

Self-compassion is acknowledged as a potential mediator in the link between attachment and depressive symptoms [33]. The results suggest an inverse relationship between positive self-compassion and depressive symptoms while indicating a positive correlation between negative self-compassion and these symptoms. Therefore, there is a variable significance observed in positive and negative self-compassion concerning depressive symptoms [9,67]. Specifically, negative self-compassion triggers negative emotions within individuals, immersing them in distressing feelings and fostering a sense of helplessness and hopelessness [37,67]. Therefore, negative self-compassion exhibits a more robust correlation with depressive symptoms in contrast to positive self-compassion. Positive self-compassion serves as a protective element against depressive symptoms by aiding individuals in self-forgiveness, understanding, and confronting experiences without self-blame [30,31]. Potentially positive self-compassion primarily serves to facilitate positive psychological outcomes rather than solely reducing negative psychological symptoms.

This research emphasizes how decreased positive self-compassion and increased negative self-compassion could play a role in averting depressive symptoms. Decreasing negative self-compassion may provide a promising avenue for interventions to mitigate depressive symptoms in college students with insecure attachment.

### 4.2. Implications

The present study reveals how attachment type affects the development of depressive symptoms through its effect on self-compassion in a college student population. These findings provide insights for mental health professionals and interventionists, offering valuable guidance for interventions targeting the prevention and treatment of depressive symptoms. Given that college students undergo significant interpersonal relationship development, interventions focusing on self-compassion could effectively promote mental health. Intervention programs designed for college students with insecure attachment could potentially alleviate their depressive symptoms by promoting an increase in positive self-compassion and a decrease in negative self-compassion. This emphasizes that intervention strategies should differ for college students exhibiting attachment anxiety versus those displaying attachment avoidance. As this study was conducted with young adults, caution should be exercised in generalizing the results to other populations. Future research could explore the relationships among insecure attachment, self-compassion, and depressive symptoms in diverse populations.

## 5. Limitations

Despite the various strengths of this study, there are still some limitations to be noted. Firstly, our data collection relied on self-report questionnaires, which are potentially susceptible to individual subjectivity and recall biases. Although we controlled for potential confounding variables in the analysis, there are still limitations to the internal validity. Further research could consider multiple data sources, such as clinical interviews, to enhance the credibility of the data. Secondly, our study’s sample predominantly comprised Chinese young adults, which could restrict the external validity of the findings. Future research could broaden the sample to encompass a more diverse population. Lastly, one limitation of cross-sectional studies is the inability to infer causal relationships. Though constrained by these limitations, this study provides valuable insights into how attachment styles impact depressive symptom and provides guidance for future research directions.

## 6. Conclusions

In conclusion, both attachment anxiety and avoidance showed a positive correlation with depressive symptoms, mediated by increased negative self-compassion and decreased positive self-compassion. Specifically, attachment avoidance predominantly impacted depressive symptoms by decreasing positive self-compassion, whereas attachment anxiety was primarily linked to depressive symptoms by intensifying negative self-compassion. Our findings offer new perspectives on how insecure attachment influences depressive symptoms in young adults, shedding light on underlying mechanisms. These findings carry implications for the development of intervention programs.

## Figures and Tables

**Table 1:** Demographic information of participants.

Variables	College Students	
	N/M	%/SD
Age	21.40	2.381
Gender		
Male	29,338	65.5%
Female	15,450	34.5%
Only-child status		
The only child	16,263	36.3%
Non-only child	28,525	63.7%
Family type		
Nuclear family (only live with parents)	28,669	64.0%
Extended family (not only live with parents)	11,266	25.2%
Not living with parents	645	1.4%
Single-parent family	2876	6.4%
Remarried family	906	2.0%
Other situations	426	1.0%
Residence		
Rural	23,643	52.8%
Towns	10,686	23.9%
Urban	10,459	23.4%

**Table 2:** Intercorrelations, means, and standard deviations for study variables.

Variable	1	2	3	4	5	6	7	8	9	10
1 Age	1									
2 Gender	0.020 **	1								
3 Siblings	0.029 **	0.053 **	1							
4 Family type	−0.060 **	0.037 **	0.030 **	1						
5 Residence	−0.058 **	0.031 **	−0.447 **	−0.061 **	1					
6 Attachment anxiety	−0.218 **	0.062 **	−0.007	0.056 **	0.034 **	1				
7 Attachment avoidance	−0.154 **	0.041 **	0.038 **	0.054 **	−0.027 **	0.604 **	1			
8 Positive SC	0.048	−0.044 **	−0.034 **	−0.039 **	0.033 **	−0.327 **	−0.419 **	1		
9 Negative SC	−0.149 **	−0.045 **	−0.052 **	−0.033 **	0.064 **	0.522 **	0.419 **	−0.135 **	1	
10 Depressive symptoms	−0.114 **	0.016 **	−0.003	0.069 **	−0.011 **	0.466 **	0.442 **	−0.363 **	0.444 **	1
*M*						14.564	30.009	22.211	15.949	4.991
*SD*						4.817	5.887	4.014	4.331	6.503

Note. ** *p* < 0.01; Positive SC = Positive Self-Compassion, Negative SC = Negative Self-Compassion, DS = Depressive symptoms.

**Table 3:** The bootstrap confidence intervals and effect sizes of the mediation model.

Mediation Paths	Estimate	*P*	95% CI
Attachment anxiety→Positive SC→DS	0.036	<0.001	[0.032, 0.040]
Attachment anxiety→Negative SC→DS	0.179	<0.001	[0.171, 0.187]
Attachment avoidance→Positive SC→DS	0.098	<0.001	[0.092, 0.104]
Attachment avoidance→Negative SC→DS	0.066	<0.001	[0.061, 0.071]
Direct effect of attachment anxiety	0.098	<0.001	[0.085, 0.111]
Direct effect of attachment avoidance	0.089	<0.001	[0.078, 0.100]

Note. Positive SC = Positive Self-Compassion, Negative SC = Negative Self-Compassion, DS = Depressive symptoms.

## Data Availability

The datasets generated during and/or analyzed during the current study are available upon reasonable request.
